# Effects of Drying Methods and Blanching on Nutrient Utilization in Black Soldier Fly Larva Meals Based on In Vitro Assays for Pigs

**DOI:** 10.3390/ani13050858

**Published:** 2023-02-26

**Authors:** Jeonghyeon Son, Seol Hwa Park, Hyun Jung Jung, Sun Jong You, Beob Gyun Kim

**Affiliations:** 1 Department of Animal Science, Konkuk University, Seoul 05029, Republic of Korea; 2 Animal Nutrition and Physiology Division, National Institute of Animal Science, Rural Development Administration, Wanju 55365, Republic of Korea; 3 Feed R&D, CJ Feed & Care, Seoul 04548, Republic of Korea

**Keywords:** black soldier fly larva meal, blanching method, drying method, in vitro assays, pigs

## Abstract

**Simple Summary:**

Black soldier fly larva meal is considered a promising protein source due to its high concentration of protein. A disinfection process is necessary to produce black soldier fly larva meal, because the black soldier fly is generally reared in food wastes or livestock manure. Drying methods include microwave drying, hot-air drying, and sun drying. Additionally, blanching as a pretreatment is also used to destroy microorganisms. However, information on the effect of these methods on nutrient utilization of black soldier fly larva meal by pigs is scarce. In the present study, the effects of drying and blanching methods on nutrient utilization were measured based on in vitro assays. Microwave-dried black soldier fly larva meal had less nitrogen digestibility than hot-air-dried black soldier fly larva meal. However, microwave-dried black soldier fly larva meal had greater nitrogen digestibility than blanched black soldier fly larva meal. The present study suggested that hot-air drying is recommended to produce black soldier fly larva meal based on the nutrient digestibility of black soldier fly larva meal for pigs.

**Abstract:**

The objective was to determine the effects of drying and blanching methods on the nutrient utilization of black soldier fly larva (BSFL; *Hermetia illucens*) meal by pigs using in vitro assays. Two-step and three-step in vitro assays were employed to simulate the gastrointestinal tract of pigs. Four BSFL meals were prepared using the following pretreatment methods: (1) microwave drying at 80 °C for 32 min, (2) hot-air drying at 60 °C for 17 h, (3) blanching for 5 min in boiling water and hot-air drying at 60 °C for 17 h, and (4) 2% citric acid solution blanching for 5 min in boiling solution and hot-air drying at 60 °C for 17 h. After the drying process, each BSFL was defatted and ground to obtain BSFL meals. The nitrogen (N) concentration in the test ingredients ranged from 8.5 to 9.4%, and the ether extract ranged from 6.9 to 11.5% on an as-is basis. The amino acid (AA) concentration in the BSFL meals ranged from 2.80 to 3.24% for Lys and 0.71 to 0.89% for Met on an as-is basis. Hot-air-dried BSFL meal had a greater in vitro ileal disappearance (IVID) of N compared with microwave-dried BSFL meal (*p* < 0.05). However, blanched BSFL meals in water or 2% citric acid solution before hot-air drying had a lower (*p* < 0.05) IVID of N compared with microwave-dried or hot-air-dried BSFL meal. Blanched BSFL meals in water or 2% citric acid solution before hot-air drying showed a lower (*p* < 0.05) in vitro total tract disappearance of dry matter and organic matter compared with microwave-dried or hot-air-dried BSFL meal. Microwave-dried BSFL meal had a lower (*p* < 0.05) IVID of indispensable AA, except for His, Lys, Met, and Phe, compared with hot-air-dried BSFL meals. However, blanched BSFL meals in water or 2% citric acid solution before hot-air drying showed a lower (*p* < 0.05) IVID of indispensable AA compared with microwave-dried or hot-air-dried BSFL meal. In conclusion, hot-air-dried BSFL meal presented greater nutrient utilization compared with microwave-dried BSFL meal for pigs. However, blanching in water or citric acid solution negatively affected the nutrient digestibility of BSFL meal based on in vitro assays.

## 1. Introduction

Fish meal and soybean meal are widely used in the swine feed industry as protein sources due to their high concentrations of digestible amino acids (AA). However, the price fluctuation of fish meal and soybean meal has increased the demand for alternative feed ingredients such as an insect protein [1]. Black soldier fly (*Hermetia illucens*) larva (BSFL) meal is considered as an alternative ingredient due to its high crude protein (CP) concentration. Research on BSFL meal as a protein source for pigs has increased [2,3,4].

Black soldier fly larva meal is potentially contaminated with microorganisms including *Salmonella*, *Escherichia coli*, *Staphylococcus aureus*, and *Bacillus cereus*, because of their rearing conditions [5,6]. Therefore, a disinfection process must precede the use of BSFL as a feed ingredient [7]. Drying is widely used to prevent microbial growth and increase the shelf life of insect protein [8]. Various drying methods have been conducted for producing insect protein, including hot-air drying, microwave drying, freeze drying, and roasting. Additionally, blanching is a pretreatment that is usually applied before the drying process to destroy microorganisms and inactivate enzymes [4,8]. These processing procedures also affect the nutritional values of BSFL meal [4,8]. However, the literature examining the influence of blanching and drying methods on the nutrient utilization of BSFL meals is scarce.

In vitro assays have been widely used to determine the nutrient utilization of feed ingredients for pigs, and the in vitro results are highly correlated with in vivo experiments [9,10,11]. To our knowledge, the effects of blanching and drying methods on the nutrient utilization of BSFL meal based on in vitro assays have not been documented. Therefore, the objective of the present study was to determine the effects of blanching and different drying methods on the nutrient utilization of BSFL meal by pigs based on in vitro assays.

## 2. Materials and Methods

### 2.1. Preparation of Black Soldier Fly Larva Meals

The BSFL were reared at 25 to 32 °C and 60 to 70% relative humidity for 15 days at a commercial black soldier fly farm (Entomo Co. Ltd., Cheongju, Republic of Korea). Dried food waste was fed to BSFL once during the whole growth duration following Control of Livestock and Fish Feed Act No. 14481 in Korea. On day 15, the BSFL were divided into four batches and dried using four different slaughter and drying procedures: (1) microwave drying at 80 °C for 32 min using a microwave (M-200, Entomo Co. Ltd., Cheongju, Republic of Korea); (2) hot-air drying at 60 °C for 17 h using a forced-air drying oven (KAPD-195D; CNT Co. Ltd., Gwangju, Republic of Korea); (3) blanching for 5 min in boiling water and hot-air drying at 60 °C for 17 h using a drying oven; and (4) 2% citric acid solution blanching for 5 min in boiling solution and hot-air drying at 60 °C for 17 h using a drying oven. The hot-air drying time was used to obtain a similar degree of dryness compared to the microwave drying process for 32 min. The blanching of BSFL for 5 min was based on the suggestion of the IPIFF [7]. After the drying process, oil was extracted from the BSFL at 45 °C using a screw-type cold press oil machine (NF-80; Karaerler Makina, Ankara, Republic of Türkiye).

### 2.2. In Vitro Procedures

A 2-step in vitro procedure was performed to measure the in vitro ileal disappearance (IVID) of dry matter (DM), nitrogen (N), and AA in the BSFL meals by simulating the digestion and absorption in the stomach and the small intestine of pigs [9,11]. In the first step of the procedure mimicking the digestion conditions of the pig stomach, 1 g of a BSFL sample was transferred into a 100 mL conical flask, and then 25 mL of sodium phosphate buffer solution (0.1 *M*, pH 6.0) and 10 mL of HCl (0.2 *M*, pH 0.7) were added to the flask. The pH was adjusted to 2.0 using 1 *M* HCl and 1 *M* NaOH solution, and 1 mL of freshly prepared pepsin solution (10 mg/mL; ≥ 250 units/mg solid, P7000, pepsin from porcine gastric mucosa; Sigma-Aldrich, St. Louis, MO, USA) was added to the flask. To inhibit microbial growth, 0.5 mL of chloramphenicol (C0378, chloramphenicol; Sigma-Aldrich, St. Louis, MO, USA) solution (5 g/L ethanol) was also added to the flask. The flasks sealed with a silicone cap were incubated in a shaking incubator (LSI-3016R; Daihan Labtech, Namyangju, Republic of Korea) at 39 °C for 6 h, and the incubator was set at 125 rpm. After the incubation, the second step of the in vitro procedure mimicking the digestion and absorption in the small intestine of pigs was performed. Firstly, 10 mL of phosphate buffer solution (0.2 *M*, pH 6.8) and 5 mL of 0.6 *M* NaOH solution were added to the flasks. Then, the pH was adjusted to 6.8 using 1 *M* HCl or NaOH solution, and 1 mL of freshly prepared pancreatin solution (50 mg/mL; 4 × USP, P1750, pancreatin from porcine pancreas; Sigma-Aldrich, St. Louis, MO, USA) was added. Thereafter, the flasks were incubated in the shaking incubator (LSI-3016R; Daihan Labtech, Namyangju, Republic of Korea) at 39 °C for 18 h, and the incubator was set at 125 rpm. After the incubation, 5 mL of 20% sulfosalicylic acid solution was added to the flasks, which were left for 30 min at room temperature to precipitate indigestible proteins. After the 30 min precipitation, undigested residues were filtered through dried glass filter crucibles (Filter Crucibles CFE Por. 2; Robu, Hattert, Germany) containing 500 mg of Celite, which helped to inhibit the plugging of the filter by the potentially gelatinous residues. The flasks were rinsed twice with 1% sulfosalicylic acid solution, and 10 mL of 95% ethanol and 10 mL of 99.5% acetone were added twice to the filter crucibles. The filter crucibles with undigested residues were dried at 80 °C for 24 h. After cooling in a desiccator for 1 h, the glass filter crucibles were weighed to calculate the IVID of DM in the BSFL meals. The undigested residues in filter crucibles were collected and analyzed for N contents to calculate the IVID of N. To determine the AA concentration of undigested residues of BSFL, the undigested residues obtained from 10 flasks were pooled within treatments to obtain a sufficient amount of residues for AA analysis. During the 2-step in vitro procedure, a blank was used to correct the DM, N, and AA contents in the residues that did not originate from the BSFL meals. 

To simulate total tract digestion and absorption, the in vitro total tract disappearance (IVTTD) of DM in the BSFL meals was measured using a 3-step in vitro technique that simulated the digestion and absorption in the stomach, the small intestine, and the large intestine of pigs [10]. The first and second steps were similar to the IVID procedure, differing in the weight of the sample, the concentration of the enzymes, and the incubation time. For the IVTTD, 0.5 g of each sample was used, and the concentrations of pepsin and pancreatic solutions were increased to 25 and 100 mg/mL, respectively, while the incubation time for steps 1 and 2 were reduced to 2 and 4 h, respectively. In the third step of the IVTTD procedure, 10 mL of 0.2 M EDTA solution was added to the samples. The pH was then adjusted to 4.8. Samples were supplemented with 0.5 mL of multi-enzyme solution (V2010, Viscozyme; Sigma–Aldrich, St. Louis, MO, USA) as a substitute for microbial enzymes and incubated in a shaking incubator for 18 h at 39 °C, and the incubator was set at 125 rpm. After the 18 h incubation, the samples were filtered, and the undigested residues were collected and dried as previously described in the IVID procedure, except that the residues were dried at 130 °C for 6 h. In addition, ash concentrations in the undigested residues were measured to calculate the IVTTD of OM in the BSFL meals. During the 3-step in vitro procedure, a blank was used to correct the DM and OM contents in the residues that did not originate from the BSFL meals. All in vitro procedures were performed in 3 replicates.

### 2.3. Chemical Analyses

The test ingredients and in vitro undigested residues were analyzed for DM [12]. The nitrogen (method 976.05) and OM (method 942.05) in the test ingredients and in vitro undigested residues were also analyzed as described in AOAC [13]. The test ingredients were also analyzed for ether extract (EE; method 920.39), ash (method 942.05), and acid detergent fiber (ADF; method 973.18), and chitin was calculated as the difference between the concentrations of ash-free ADF and ADF-linked protein [3,13]. The AA concentrations of the test ingredients and in vitro undigested residues were determined by ion-exchange chromatography using post-column derivatization with ninhydrin. Before the analysis, samples were liberated from the protein by hydrolysis with 6 *N* HCl for 24 h at 110 °C, as described in AOAC (method 982.30) [13]. Methionine and cystine were analyzed as methionine sulfone and cysteic acid after cold performic acid oxidation overnight before hydrolysis. All chemical analyses were performed in duplicate.

### 2.4. Calculations and Statistical Analyses

The IVID or IVTTD of DM was calculated using the following equation from Choi et al. [9]:

IVID or IVTTD of DM (%) = [DM_TI_ − (DM_UR_ − DM_Blank_)] ÷ DM_TI_ × 100

where DM_TI_ (g) is the amount of test ingredients on a DM basis, DM_UR_ (g) is the amount of undigested residue on a DM basis after in vitro assays, and DM_Blank_ (g) is the amount of DM residue on a DM basis after in vitro digestion procedures in the blank.

After the 2-step in vitro assay, the undigested residues and celite were collected, weighed, and analyzed for N and AA. Then, the IVID of N or AA was calculated using the following equation modified from Kim et al. [14]:

IVID of N or AA (%) = [(DM_TI_ × C_TI_) − {(DM_UR_ × C_UR_) − (DM_Blank_ × C_Blank_)}] ÷ (DM_TI_ × C_TI_) × 100

where C_TI_, C_UR_, and C_Blank_ (g) are the N or AA concentration (%) expressed on a DM basis in the test ingredients, undigested residue after 2-step in vitro assays, and blank, respectively.

After the 3-step in vitro assay, the undigested residues and celite were collected, weighed, and analyzed for OM. Then, the IVTTD of OM was calculated using the following equation:

IVTTD of OM (%) = [(DM_TI_ × OM_TI_) − {(DM_UR_ × OM_UR_) − (DM_Blank_ × OM_Blank_)}] ÷ (DM_TI_ × OM_TI_) × 100

where OM_TI_, OM_UR_, and OM_Blank_ (g) are the OM concentration (%) expressed on a DM basis in the test ingredients, undigested residue after 3-step in vitro assays, and blank, respectively.

Experimental data were analyzed using the GLM procedure of SAS (SAS Inst. Inc., Cary, NC, USA). The test ingredient was included as a fixed variable in the model. The least squares means were calculated for the IVID of DM, N, and AA and the IVTTD of DM and OM for each test ingredient and compared using the PDIFF option with Tukey’s adjustment. Each flask was considered as the experimental unit for the in vitro disappearance of nutrients, and a pooled sample from 10 flasks was considered as the experimental unit for the IVID of AA. The statistical significance was declared at *p* < 0.05.

## 3. Results

The ash and N concentrations in the four sources of BSFL meal ranged from 12.9 to 22.0% and from 8.5 to 9.4% (as-is basis), respectively (Table 1). The EE in the test ingredients ranged from 6.9 to 11.5%, and the chitin in the test ingredients ranged from 4.3 to 7.2%. The analyzed Lys concentrations in the four BSFL meals ranged from 2.80 to 3.24%, and the Met concentrations ranged from 0.71 to 0.89%.

Hot-air-dried BSFL meal had a greater IVID of N compared with microwave-dried BSFL meal (*p* < 0.05; Table 2). However, blanched BSFL meal in water or 2% citric acid solution before hot-air drying had a lower IVID of N (*p* < 0.05) compared with microwave-dried or hot-air-dried BSFL meal. Blanched BSFL meal in water or 2% citric acid solution before hot-air drying showed a lower IVTTD of DM (*p* < 0.05) and OM (*p* < 0.05) compared with microwave-dried or hot-air-dried BSFL meal. The IVTTD of OM in blanched BSFL meal in 2% citric acid solution before hot-air drying was greater than that in blanched BSFL meal in water before hot-air drying. 

The IVID of Arg, Ile, Leu, and Thr in microwave-dried BSFL meal was lower (*p* < 0.05) than that in hot-air-dried BSFL meal without blanching but greater (*p* < 0.05) than that in blanched BSFL meals in water or 2% citric acid solution before hot-air drying (Table 3). The IVID of His, Lys, Met, and Phe in microwave-dried or hot-air-dried BSFL meal was greater (*p* < 0.05) compared with that in blanched BSFL meals in water or 2% citric acid solution before hot-air drying. The IVID of Ile in blanched BSFL meal in water before hot-air drying was greater (*p* < 0.05) than that in blanched BSFL meal in 2% citric acid before hot-air drying.

## 4. Discussion

Black soldier fly larvae are regarded as a promising protein source due to their high N concentrations and the high gain-to-feed ratio of fly production [8]. Considering the rearing conditions of black soldier fly, BSFL meals may be contaminated with microbial pathogens, and thus a disinfection process is necessary to produce safe feed ingredients. Various heat treatments are used to reduce the microbial risk and increase the shelf life of BSFL meals [7]. However, these heat treatments potentially affect the physicochemical properties to varying degrees, depending on the drying temperature and time. In a previous study, microwave drying affected the AA profiles and increased the size of protein particles in BSFL meal [15]. In addition, hot-air drying can stimulate the denaturation of proteins, lipid oxidation, and melanization [16,17]. Blanching prior to drying can also cause the alteration of the nutrient composition [18] and texture of insect protein [8]. These changes in physicochemical properties might alter the nutrient utilization of BSFL meals, which can be evaluated using in vitro assays. To our knowledge, however, the effects of blanching and drying methods on the in vitro disappearance of nutrients in BSFL meal for pigs have not been documented. In the present work, the effects of blanching and drying methods on the nutrient utilization of BSFL meals by pigs were determined using an in vitro assay.

The analyzed chemical compositions of BSFL meals in the current work were mostly within the ranges of previous studies [2,19,20]. However, the ash concentrations of the four sources of BSFL meal were higher than the values in previous studies [2,19,20]. In addition, the EE concentrations of the BSFL meals, except for the BSFL meal blanched in 2% citric acid solution before hot-air drying, were lower than in previous studies [2,19,20]. The reason for this discrepancy may have been the different rearing conditions of the BSFL and the processing methods for euthanizing, drying, and oil extraction [4,21]. Although the four BSFL meals were reared under the same environmental conditions and fed the same diets, the ash concentrations in the four BSFL meals varied in the present study. Bawa et al. [22] reported that the ash concentration in microwave-dried crickets (*Henicus whellani*) was greater than that in hot-air-dried crickets. This finding is consistent with the results from the present study. Mshayisa et al. [23] reported that the addition of acid to the solution decreased the pH of the solution and the protein solubility, which facilitated the layer separation of BSFL meal during the oil extraction process and the removal of the mineral fraction. Similarly, Soetemans et al. [24] also reported that the use of organic acids improved the fractionation of the BSFL into protein factions. During blanching, potential losses of protein are minimized at pH 5, which was achieved in 2% citric acid in the present work. In terms of EE, the effects of the drying and blanching methods were also inconsistent. In a previous study [8], scalding in boiling water before hot-air drying for 4 min increased the EE concentration in BSFL meal. In another study [25], however, water blanching before freeze drying did not change the lipid content of the BSFL meal, in agreement with the present study. Although it is uncertain why water blanching did not affect the EE concentration of BSFL meal, the deviations in the EE concentrations may have been due to factors including the rearing conditions, oil extraction process, and storage conditions. The reason for the high ADF and chitin in BSFL meals blanched in water or 2% citric acid solution was unclear. Although examining a different species, Fox et al. [26] reported that water blanching before drying increased the chitin concentration in shrimp head meal. Water blanching before hot-air drying increased the N and indispensable AA concentrations in BSFL meals. This observation was consistent with a previous study [18].

The in vitro disappearance of nutrients in BSFL meals in the present work was within the range of previously reported values [27,28,29,30], but the IVID of N in BSFL meals blanched in water or 2% citric acid before hot-air drying was slightly lower than that in previous studies [27,29,30]. The decreased IVID of N in BSFL meals after water or 2% citric acid blanching followed by hot-air drying was likely due to the high concentration of chitin in these BSFL meals. Chitin is poorly digested by the endogenous digestive enzymes of pigs and is negatively correlated with nutrient utilization [3]. Additionally, chitin is a major fiber fraction in the exoskeleton of insects and contains chitinous N, which is one of the major reasons for the overestimation of CP concentrations in insect meals [23]. The higher IVID of N in hot-air-dried BSFL meals than microwave-dried BSFL meal in the present work was in agreement with previous findings [15]. Shorstkii et al. [31] also reported that microwave drying denatured and polymerized the protein particles of BSFL, and it was harder for the digestive enzymes to digest the denatured proteins. The effects of blanching on the IVID of N varied among the studies. Manditsera et al. [32] reported that boiling for 30 min did not affect the in vitro protein hydrolysis of roasted beetle (*Eulepida mashona*) and cricket (*Henicus whellani*). However, Zielińska et al. [33] reported that 10 min boiling improved the degree of hydrolysis in mealworm (*Tenebrio molitor*) and desert locust (*Schistocerca gregaria*), and Singh et al. [18] reported that blanching improved the in vitro CP digestibility of house cricket (*Acheta domesticus*). This discrepancy may be attributed to the differences in species, in vitro procedures, and boiling times.

The ranking of the IVID of AA among the test ingredients was similar to that of the IVID of N for pigs. This finding indicated that the IVID of N in BSFL meal was positively correlated with the IVID of AA in BSFL meal. In a previous in vitro experiment simulating the human gastrointestinal tract conducted by Huang et al. [15], the in vitro digestibility of all AA in hot-air-dried BSFL meal was higher than that in microwave-dried BSFL meal. This finding was consistent with the IVID of some AA in the present study and indicates that BSFL meal dried using a microwave is harder to digest than hot-air-dried BSFL meal. Along with N digestibility, water or 2% citric acid blanching also decreased the IVID of indispensable AA in BSFL meal. To the best of our knowledge, the in vitro AA digestibility in BSFL meal for pigs was reported herein for the first time. Like the IVID of N, the decreased IVID of AA after blanching was likely due to the increased chitin concentration. Additionally, chitin may form aggregates with digestive enzymes and decrease the IVID of AA. The addition of 2% citric acid during the blanching before hot-air drying decreased the IVID of Ile. The reason for this decrease in the IVID of Ile after 2% citric acid solution blanching prior to hot-air drying was not clear, and further research is needed to clarify the effect of blanching in citric acid on the IVID of Ile.

## 5. Conclusions

In conclusion, the addition of citric acid during blanching before hot-air drying increased the N concentration and decreased the ash concentration of BSFL meal. Hot-air-dried BSFL meal presented greater nutrient utilization by pigs compared with microwave-dried BSFL meal. However, blanching in water or citric acid solution negatively affected the nutrient digestibility of BSFL meal for pigs based on in vitro assays.

## Figures and Tables

**Table 1:** Analyzed chemical compositions of four black soldier fly larva meals with various drying and blanching methods, as-is basis ^1^.

Item, %	Drying Method:	Microwave	Hot-Air	Hot-Air	Hot-Air
	Blanching:	-	-	Water	2% Citric Acid Solution
Dry matter		96.8	96.1	97.4	97.6
Organic matter		74.8	80.6	81.8	84.7
Ash		22.0	15.5	15.6	12.9
Nitrogen		8.5	8.9	9.4	9.1
Ether extract		6.9	7.4	7.4	11.5
Acid detergent fiber		10.8	12.7	18.8	17.6
Chitin ^2^		4.3	4.8	7.1	7.2
Indispensable amino acids					
Arg		2.43	2.55	2.79	2.56
His		1.43	1.43	1.71	1.46
Ile		2.14	2.31	2.48	2.11
Leu		3.32	3.62	3.93	3.57
Lys		2.80	2.96	3.24	2.88
Met		0.71	0.86	0.89	0.88
Phe		1.99	2.28	2.42	2.10
Thr		1.96	2.12	2.23	2.09
Val		4.39	4.27	5.49	5.14
Dispensable amino acids					
Ala		3.39	3.82	3.56	3.43
Asp		4.42	4.76	5.11	4.85
Cys		0.38	0.42	0.38	0.36
Glu		6.42	6.06	6.59	6.39
Gly		2.69	2.65	3.08	2.89
Pro		3.15	3.08	3.45	3.23
Ser		2.19	2.23	2.49	2.32
Tyr		2.98	3.03	3.64	3.28

^1^ Samples were analyzed in duplicates. ^2^ Chitin (%) = ash-free acid detergent fiber (%)—acid detergent fiber-linked protein (%).

**Table 2:** In vitro ileal disappearance (IVID) of dry matter (DM) and nitrogen (N) and in vitro total tract disappearance (IVTTD) of DM and organic matter (OM) in four black soldier fly larva meals with various drying and blanching methods for pigs ^1^.

Item, %	Drying Method:	Microwave	Hot-Air	Hot-Air	Hot-Air	SEM	*p*-Value
	Blanching:	-	-	Water	2% Citric Acid Solution		
IVID of DM		84.6 ^a^	84.8 ^a^	80.1 ^b^	82.9 ^ab^	0.6	0.003
IVID of N		84.4 ^b^	86.7 ^a^	81.3 ^c^	82.5 ^c^	0.3	<0.001
IVTTD of DM		90.4 ^a^	89.6 ^a^	85.0 ^b^	84.5 ^b^	0.3	<0.001
IVTTD of OM		85.7 ^b^	86.6 ^a^	80.6 ^d^	81.5 ^c^	0.1	<0.001

SEM = standard error of the means. ^1^ Each least squares mean represents three observations. ^a–d^ Least squares means with a row without a common superscript letter differ (*p* < 0.05).

**Table 3:** In vitro ileal disappearance of amino acids in four black soldier fly larva meals with various drying and blanching methods for pigs ^1^.

Item, %	Drying Method:	Microwave	Hot-Air	Hot-Air	Hot-Air	SEM	*p*-Value
	Blanching:	-	-	Water	2% Citric Acid Solution		
Indispensable amino acids							
Arg		94.4 ^b^	96.5 ^a^	90.7 ^c^	90.8 ^c^	0.3	<0.001
His		93.8 ^a^	95.6 ^a^	89.6 ^b^	87.7 ^b^	0.5	<0.001
Ile		91.5 ^b^	94.0 ^a^	87.8 ^c^	85.1 ^d^	0.4	<0.001
Leu		90.7 ^b^	92.8 ^a^	85.7 ^c^	86.5 ^c^	0.4	<0.001
Lys		93.6 ^a^	92.3 ^a^	89.9 ^b^	87.8 ^c^	0.4	<0.001
Met		88.8 ^a^	91.4 ^a^	81.9 ^b^	83.2 ^b^	0.8	<0.001
Phe		89.1 ^a^	89.7 ^a^	85.0 ^b^	83.1 ^b^	0.9	0.002
Thr		90.8 ^b^	92.7 ^a^	86.7 ^c^	87.7 ^c^	0.4	<0.001
Dispensable amino acids							
Ala		92.5 ^b^	94.2 ^a^	88.3 ^d^	89.7 ^c^	0.3	<0.001
Asp		91.1 ^b^	93.2 ^a^	86.8 ^d^	88.5 ^c^	0.3	0.005
Cys		82.5 ^a^	81.3 ^ab^	77.3 ^bc^	75.3 ^c^	1.1	<0.001
Glu		94.2 ^a^	94.5 ^a^	91.3 ^c^	92.3 ^b^	0.2	<0.001
Gly		87.2 ^a^	88.7 ^a^	81.1 ^b^	82.4 ^b^	0.6	<0.001
Pro		94.3 ^a^	94.3 ^a^	90.3 ^b^	90.6 ^b^	0.3	<0.001
Ser		90.6 ^a^	91.8 ^a^	87.0 ^b^	87.8 ^b^	0.4	<0.001
Tyr		93.1 ^a^	94.7 ^a^	89.1 ^b^	88.3 ^b^	0.5	<0.001

SEM = standard error of the means. ^1^ Each least squares mean represents three observations. ^a–d^ Least squares means with a row without a common superscript letter differ (*p* < 0.05).

## Data Availability

The data presented in the current work are available.
